# Dual oncogenic and tumor suppressor roles of the promyelocytic leukemia gene in hepatocarcinogenesis associated with hepatitis B virus surface antigen

**DOI:** 10.18632/oncotarget.8613

**Published:** 2016-04-06

**Authors:** Yih-Lin Chung, Mei-Ling Wu

**Affiliations:** ^1^ Department of Radiation Oncology, Koo Foundation Sun-Yat-Sen Cancer Center, Taipei, Taiwan; ^2^ Department of Pathology and Laboratory Medicine, Koo Foundation Sun-Yat-Sen Cancer Center, Taipei, Taiwan

**Keywords:** hepatitis B virus, hepatocarcinogenesis, PML, tumor suppressor, oncogene

## Abstract

Proteasome-mediated degradation of promyelocytic leukemia tumor suppressor (PML) is upregulated in many viral infections and cancers. We previously showed that *PML* knockdown promotes early-onset hepatocellular carcinoma (HCC) in *hepatitis B virus surface antigen* (*HBsAg)*-transgenic mice. Here we report the effects of PML restoration on late-onset HBsAg-induced HCC. We compared protein expression patterns, genetic mutations and the effects of pharmacologically targeting PML in *wild-type*, *PML^−/−^*, *PML^+/+^HBsAg^tg/o^* and *PML^−/−^HBsAg^tg/o^* mice. *PML^−/−^* mice exhibited somatic mutations in DNA repair genes and developed severe steatosis and proliferative disorders, but not HCC. *PML^−/−^HBsAg^tg/o^* mice exhibited early mutations in cancer driver genes and developed hyperplasia, fatty livers and indolent adipose-like HCC. In *PML^+/+^*HBsAg**-transgenic mice, HBsAg expression declined over time, and HBsAg-associated PML suppression was concomitantly relieved. Nevertheless, these mice accumulated mutations in genes contributing to oxidative stress pathways and developed aggressive late-onset angiogenic trabecular HCC. PML inhibition using non-toxic doses of arsenic trioxide selectively killed long-term HBsAg-affected liver cells in *PML^+/+^HBsAg^tg/o^* mice with falling HBsAg and rising PML levels, but not normal liver cells or early-onset HCC cells in *PML^−/−^HBsAg^tg/0^* mice. These findings suggest dual roles for PML as a tumor-suppressor lost in early-onset HBsAg-induced hepatocarcinogenesis and as an oncogenic promoter in late-onset HBsAg-related HCC progression.

## INTRODUCTION

Hepatocellular carcinoma (HCC) is one of the most common deadly tumors worldwide [1]. HCC can result from chronic viral hepatitis, mainly after hepatitis B virus (HBV) or hepatitis C virus (HCV) infections. HCC may also result from alcoholic liver disease, aflatoxin exposure, and metabolic diseases, such as hereditary hemochromatosis, alpha-1 antitrypsin deficiency or non-alcoholic fatty liver disease [2]. Nevertheless, chronic HBV infections remain the main etiology of HCC in Asia. The risk of HCC development for HBV carriers is 223-fold higher than for non-carriers [3].

HBV has a partially double-stranded, 3.2-kb genome. It is a non-cytopathic hepatotropic DNA virus classified as a member of the *hepadnaviridae* family [4][5][6]. The 3.2-kb genome can be unidirectionally transcribed from four promoters to produce various HBV proteins. The first promoter, called the basal core, produces precore (HBeAg) and pregenomic (HBV-core and HBV-polymerase [Pol] proteins). The second promoter, SPI, produces a large HBV surface antigen (HBsAg). The third promoter, SPII, produces intermediate and small HBsAgs. The fourth promoter, X, produces the X protein, whose function is not fully understood [7][8][9]. The HBV genome replication cycle in the host can be divided broadly into three phases. First, a partially double-stranded, relaxed circular DNA (RC-DNA) is produced; second, the RC-DNA is converted to a covalently closed circular DNA (cccDNA); and third, the cccDNA is transcribed by the cellular RNA polymerase II to produce pregenomic RNA (pgRNA) and subgenomic RNA. In the active HBV replication state, the HBV-core protein is transcribed from the pgRNA; then, HBV-core selectively packages pgRNA into progeny capsids. Next, the pgRNA is reverse-transcribed by the co-packaged Pol to produce new RC-DNA. Individual copies of mature RC-DNA are enveloped by HBsAg to form progeny virions that are released into the circulation. Clinically, detection of HBsAg is the hallmark of an HBV infection; detection of HBeAg, which is processed from the precore protein, is a marker of infectivity; and detection of circulating HBV DNA is an indicator of active replication [7]. HBsAg becomes undetectable in most patients four to six months after acute HBV exposure. In less than 1% of patients infected with HBV, HBsAg persists for more than six months, indicating a chronic infection and integration of the HBV genome into the host chromosome [8][9]. In an inactive carrier state, the clearance rate of HBsAg is slow (∼0.5% per year), regardless of the expression levels of HBV-derived RNA and DNA and other viral products [10].

Recent studies have shown that, upon viral entry, sensors and/or receptors in the infected host cells can detect virus-derived RNAs, DNAs, and proteins. These sensors activate intracellular signaling pathways that induce inflammatory cytokines, chemokines, interferons, gene transcription, and protein modifications, which exert innate and adaptive immune responses against viral replication [11]. When the virus cannot be eradicated, the accumulated viral proteins will induce endoplasmic reticulum (ER) stress and unfolded-protein responses that inhibit protein synthesis, induce protein degradation, trigger apoptosis, and facilitate cell transformation [12][13]. Although there is significant evidence for the effects of HBsAg-induced ER stress on hepatocarcinogenesis in HBV carriers [13][14], no specific genes are universally altered by the HBsAg-induced oxidative damage and HBV DNA integration. We hypothesized that hepatocarcinogenesis involves interplay between HBV and host hepatocytes. Therefore, the present study aimed to identify host tumor suppressors and/or oncogenic proteins that interact with HBsAg during HBV-associated pathogenesis. Identifying such proteins might facilitate the development of treatments for HCC.

## RESULTS

### Spatiotemporally reciprocal negative interactions between human PML and HBsAg during human HBV-related pathogenesis

**Figure 1:**
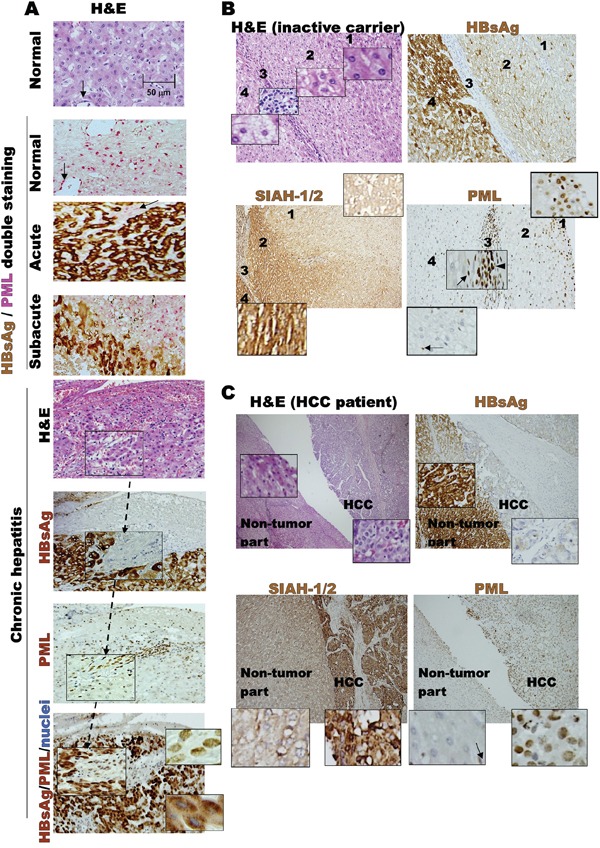
Dynamic interaction between HBV and host factors during chronic HBV-related pathogenesis **A.** Liver biopsies from initial normal state, acute HBV infection and chronic HBV hepatitis patients. Double-stainings of cytoplasmic HBsAg (brown) and nuclear PML (pink) are shown at different disease stages, exhibiting mutual exclusion and reciprocal negative expression patterns. Immunohistochemistry of chronic hepatitis was counterstained with Mayer's Hematoxylin (blue) for nuclei visualization in tissue sections. PML-positive HBsAg-negative sinusoidal endothelial cells (arrow), and mononuclear infiltrating cells in chronic hepatitis or septum lymphocytes (arrowhead) in the HBV carrier are internal staining controls. **B.** Tissues from an HBV carrier with zones 1, 2, 3 (the septum) and 4 in the same liver section exhibiting differential expression patterns of SIAH-1/2, PML and HBsAg, as well as distinct changes in fat distribution. **C.** Expression patterns of SIAH-1/2, PML and HBsAg in tissues from patient with HBV-related HCC, compared to those of the surrounding non-tumor liver.

Ubiquitin-mediated degradation appears to be the common mechanism accounting for loss of the tumor suppressor promyelocytic leukemia (PML) in virus infection and cancer [15][16]. The ubiquitin E3-ligases, SIAH-1 and -2 (SIAH-1/2), are implicated in tumorigenesis through physical interaction with PML triggering its degradation [17][18][19][20]. In addition, our earlier study in 155 HBV-infected patients demonstrated that suppression of PML persisted until HBsAg expression was gradually down-regulated [21]; and our analysis of each HBV component effect by transfection assays revealed that HBsAg induced proteasome-mediated PML degradation [22]. These findings led us to ask whether the tumorigenesis of HBsAg was associated with SIAH-1/2-mediated PML loss [23]. Therefore, in order to examine the interactive expression patterns of PML, SIAH-1/2 and HBsAg during the HBV-related pathogenesis, the liver tissue arrays from our previously studied 155 HBV-infected patients were further stratified by their clinical phases of HBV infection for immunohistochemistry [21][24]. Clinically, chronic HBV pathogenesis can be subdivided into several phases: the immune tolerance (acute) phase, the immune clearance (chronic hepatitis) phase, the inactive carrier phase and HCC formation. To show expression patterns of HBV surface antigen (HBsAg) and the host growth-regulated proteins, PML and SIAH-1/2, liver tissue arrays from different phases were immunostained with anti-HBsAg, anti-PML and anti-SIAH-1/2. We found that, in acute HBV infections, diffuse, strong HBsAg expression correlated with complete suppression of PML expression (Figure 1A). Conversely, in the subsequent phases of chronic hepatitis, inactive carrier and HCC development, a gradual-to-total disappearance or downregulation of HBsAg was accompanied by re-appearance of nuclear PML (Figures 1A, 1B and 1C). In contrast, SIAH-1/2 expression did not concordantly change in response to the dynamics of HBsAg or inversely correlate with fluctuating PML expression (Figures 1B and 1C). Thus, the consistent spatiotemporal findings of mutual exclusion (Figure 1A) and reciprocal negative interaction (Figure 1B) between HBsAg and PML during the whole course of HBV-induced pathogenesis and HCC development prompted us to investigate whether different biological consequences progress from HBsAg^extensive^PML_suppression_ to HBsAg^lost^PML_re-appearance_ conditions (Figure 1C).

### Phenotypes and pathogenesis of *HBsAg*-transgenic mice with or without *PML* deletion

**Figure 2:**
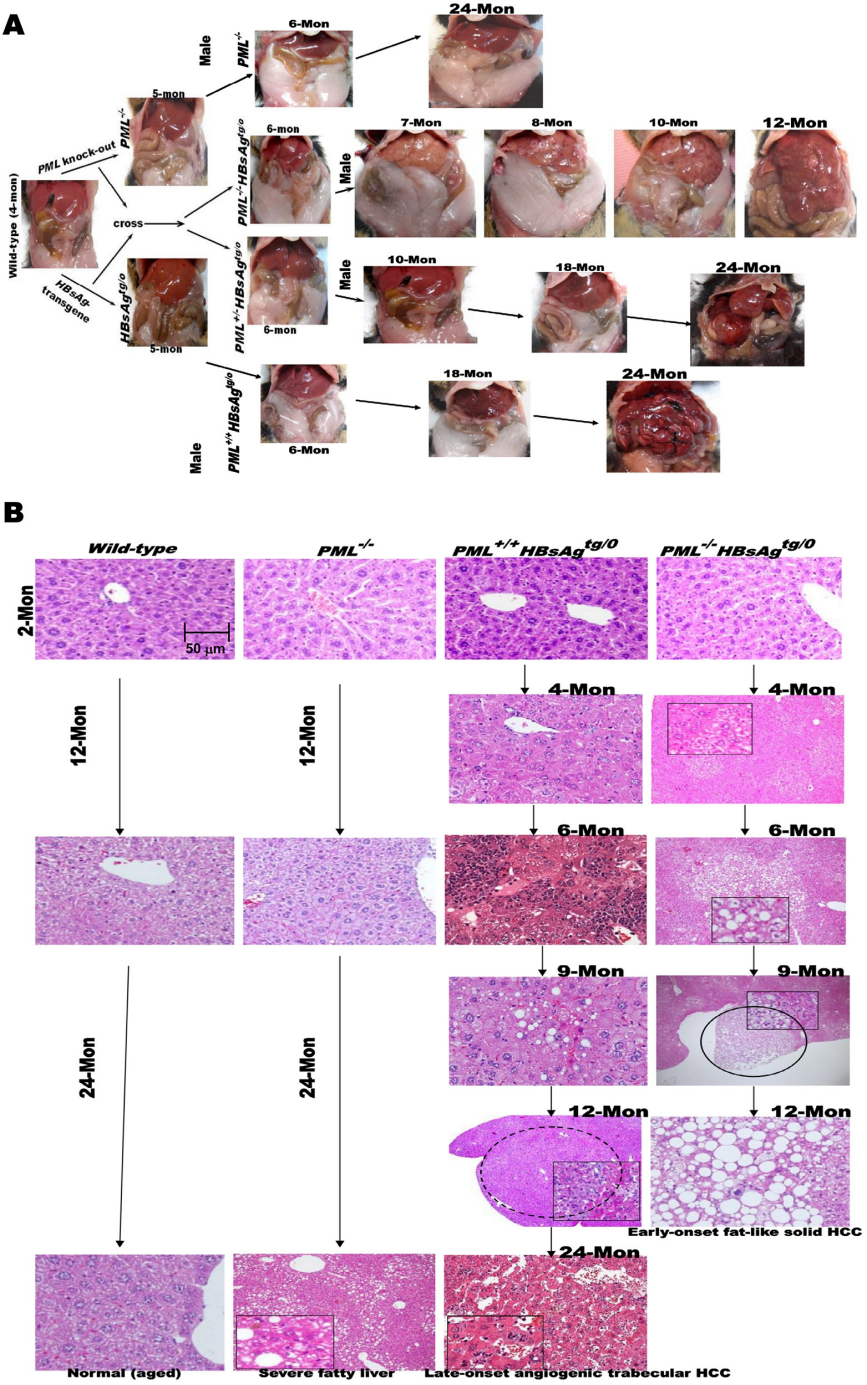
Phenotypes and pathogenesis of liver-specific HBsAg-transgenic mice with or without PML deletion **A.** Open abdominal cavities of *PML*-knockout mice (129/SV-*Pml*^t*m/Ppp*^) crossed with liver-specific *HBsAg*-transgenic mice (C57BL/6J-Tg(Alb1HBV)44Bri/J), and of offspring crossed to generate different genotypes, including *wild-type, PML^−/−^*, *PML^+/+^HBsAg^tg/0^*, *PML^+/−^HBsAg^tg/0^* and *PML^−/−^HBsAg^tg/0^*. Each genotype shows gross changes over time in the liver and body fat masses with HCC progression. Once liver tumors develop, the body fat mass seems to be consumed, a sign of cachexia. Mon = months. **B.** Representative histological images of liver sections from *wild-type*, *PML^−/−^*, *PML^+/+^HBsAg^tg/0^* and *PML^−/−^HBsAg^tg/0^* mice sacrificed at 2 -24 months of age. Adenomas are indicated by circles.

**Figure 3:**
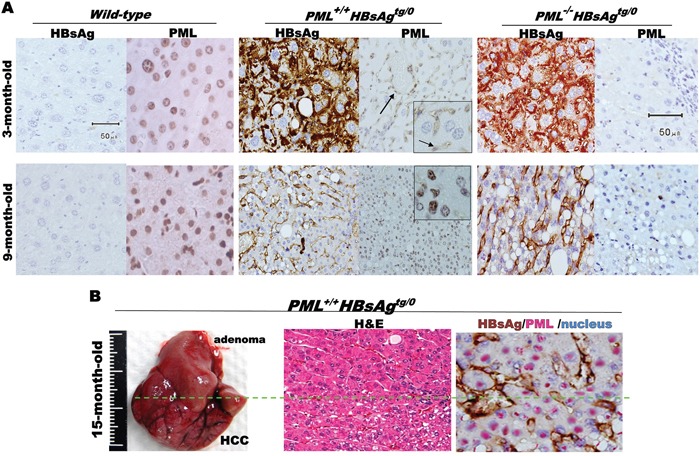
Mutually exclusive interactive patterns between PML and HBsAg in HBsAg-transgenic mice **A.** Immunohistochesmistry with anti-PML and/or anti-HBsAg antibodies counterstained with Mayer's Hematoxylin (blue) for visualization of nuclei in liver sections from *wild-type*, *PML^+/+^HBsAg^tg/0^* and *PML^−/−^HBsAg^tg/0^* mice at 3-9 months of age. PML-positive HBsAg-negative sinusoidal endothelial cells (arrow) highlighted as internal positive staining controls. **B.** Liver of *PML^+/+^HBsAg^tg/0^* mouse with late-onset HCC (left) at 15 months of age and double-staining of tissue sections (middle and right) showing a mutual exclusion pattern between the fading cytoplasmic or membrane HBsAg (brown) and the restored nuclear PML (pink).

In order to examine the influence of PML on HBsAg-driven hepatocarcinogenesis, we crossed *PML^−/−^* knockout mice with a liver-specific, *HBsAg*-transgenic mice to generate *wild-type*, *PML^−/−^*, *PML^+/+^*HBsAg*^tg/0^*, *PML^+/−^*HBsAg*^tg/0^* and*PML^−/−^*HBsAg*^tg/0^* mice with the same genetic backgrounds (Figure 2A). The *PML^−/−^* mice showed body fat accumulation, pale livers, but no liver tumor development even at two years of age. Complete loss of PML in the *HBsAg*-transgenic mice (*PML^−/−^*HBsAg*^tg/0^*) conferred a strong growth advantage and altered lipid metabolism, predisposing the mice to obesity and accelerating the development of multiple hyperplasial foci and large fatty adenomas, which appeared at 12-20 weeks of age, followed by formation of solid adipose-like HCCs by one year of age. In contrast, almost all of the *PML^+/+^HBsAg^tg/0^* mice exhibited a protracted two-year course of hepatocarcinogenesis, which started with ground-glass hepatocytes and progressed to steatohepatitis, dysplastic adenomas, and finally, angiogenic trabecular HCC formation (Figure 2B). Interestingly, PML appeared to act in a haploinsufficient manner in *PML^+/−^HBsAg^tg/0^* mice that showed mixed phenotypic features of *PML^−/−^HBsAg^tg/0^* and *PML^+/+^HBsAg^tg/0^* mice. Moreover, PML and HBsAg patterns in the liver cells of *HBsAg*-transgenic mice recapitulated the mutual exclusion and reciprocal negative interaction observed in chronic human HBV-infected liver cells (Figures 1 and 3A). Although the *HBsAg* transgene has been found to be intact at the integrated site and can be reactivated, progressive loss of HBsAg is a characteristic common to human and mouse HBV-related pathogenesis [10][25]. However, we found that when HBsAg decayed over time, the reappearance of the bona fide tumor suppressor PML did not retard the late-onset progression of HCC in *PML^+/+^HBsAg^tg/0^* mice (Figure 3B).

### Mutation landscape of *HBsAg*-transgenic mice with or without *PML* deletion

**Figure 4:**
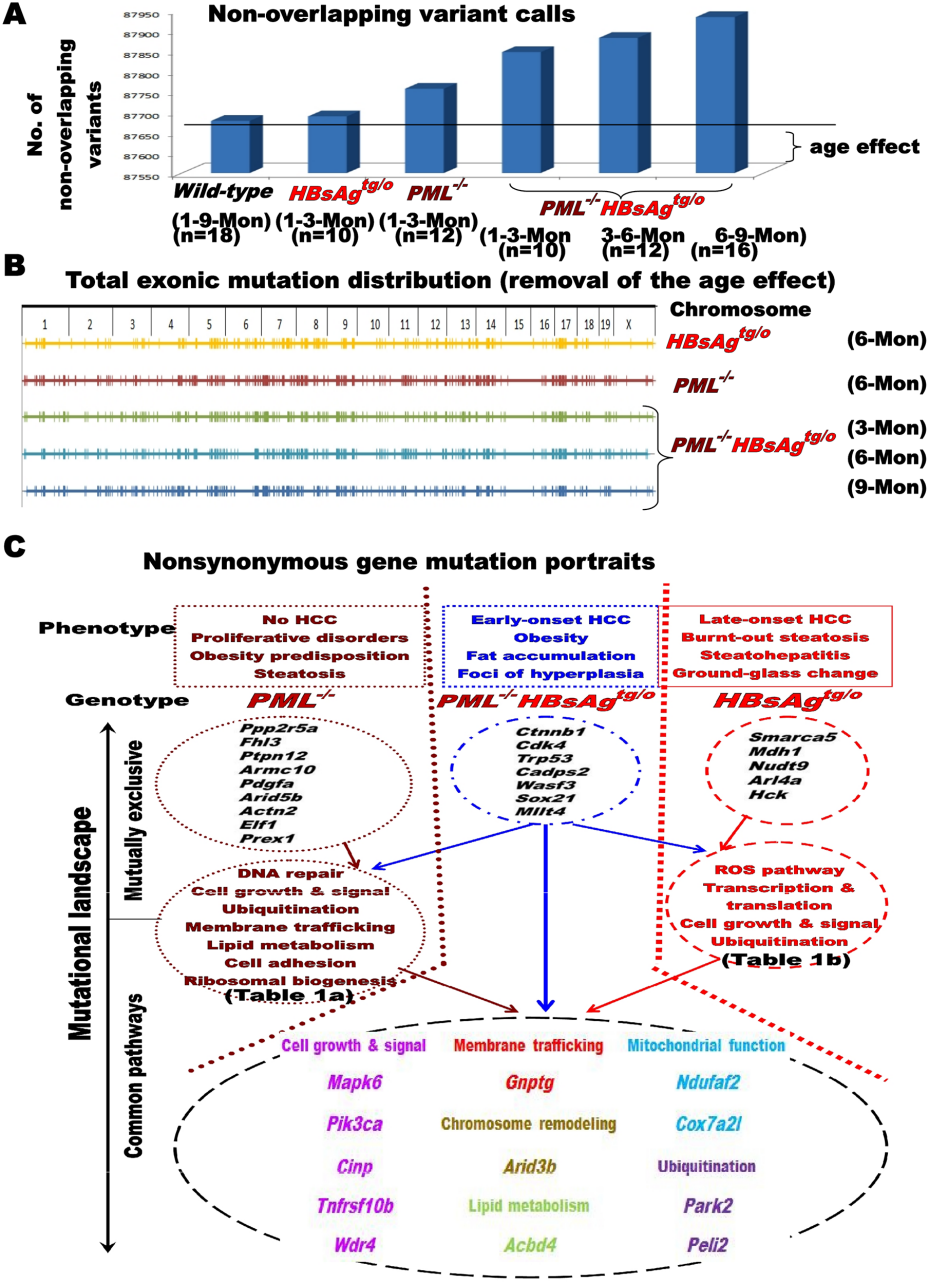
Impact of PML on the mutational landscape of HBsAg-related HCC **A.** Effect of age on gene mutations in *wild-type, PML^+/+^HBsAg^tg/0^* and *PML^−/−^HBsAg^tg/0^* mice. **B.** Representative chromosome maps showing the effects of *PML* deletion and/or HBsAg expression on genomic stability after removing the age effect. **C.** Diagram illustrating mutually exclusive and co-occurring, nonsynonymous gene mutations in *PML^−/−^*, *PML^−/−^HBsAg^tg/0^* and *PML^+/+^HBsAg^tg/0^* mice, as well as their functions. Enriched genes were categorized by gene ontology analysis. Known and established gene alterations (>50% mutation rates) are listed and grouped for each genotype to facilitate correlations with the representative phenotype.

Given that PML is a genome guardian [21][22][26], we performed whole-exome sequencing of DNA derived from liver cells of *wild-type, PML^−/−^, PML^+/+^HBsAg^tg/o^*, and *PML^−/−^HBsAg^tg/o^* mice, with or without early-onset or late-onset liver tumors (Figure 4 and Table 1). Whole exome sequences were screened for mutations in 10-18 mice of each genotype at different ages to identify unique, mutually exclusive, and co-occurring genes pertinent to HBsAg-induced HCC with or without *PML* deletion (*PML^−/−^*). Except for the mutated exonic sequences of *PML* in the *PML*-knockout mice, confirmed to match those previously reported [27], there were no differences in exonic DNA variants or polymorphisms at one month of age between different genotypes, indicating homogeneous genetic background at the baseline. Furthermore, no *HBsAg* transgene had integrated into the exomes (Supplementary file 1). The cumulative DNA variants, found in more than 50% of the wild-type mice that did not develop adenoma and HCC at two years of age, were regarded as age-related mutations (Figure 4A). By comparing the variants of genetically engineered mice with those of age-matched, wild-type mice, we removed the age effect on nonspecific gene mutations. At 6 months of age, the *PML^−/−^*, *PML^−/−^HBsAg^tg/0^* and *PML^+/+^HBsAg^tg/0^* mutation maps show low mutation rates (or cold areas) on chromosomes 15 and X; hypermutational regions (or kataegis) are visible on chromosomes 4-9, 11-14, and 17 (Figure 4B). We then identified the nonsynonymous mutation landscape in each genotype (Figure 4C).

**Table 1:** Common gene mutations compared between *PML^−/−^ and PML^−/−^ HBsAg^tg/0^* and between *HBsAg^tg/0^* and *PML^−/−^ HBsAg^tg/0^*

(a) Co-occurring gene alterations in *PML^−/−^ and PML^−/−^HBsAg^tg/0^mice*						
Cell growth & signal	DNA repair	Ubiquitination	Membrane trafficking	Lipid metabolism	Cell adhesion	Ribosomal biogenesis
*Ppp1r13l*	*Pcna*	*Usp2*	*Pld5*	*Ugt1a9*	*Itgad*	*Rpl32*
*Fat2*	*Ercc1*	*Sae1*	*Vps52*	*Nr1d2*	*Itgb2l*	*Mrpl55*
*Edaradd*	*Fancc*	*Ube4a*	*Cpnel*	*Glud1*	*Fat2*	*Rrs1*
*Fam59a*	*Ybx1*	*Siah1a*	*Gpi1*			
*Loxl4*	*Rest*	*Rnf167*				
*Ptprg*	*Purb*	*Cbl*				
*Pla2g4e*	*Ncapd2*					
*Ybx1*	*Cep164*					
*Cdk6*	*Rrm2*					
*Nop2*	*Smc5*					
*Relb*						
*Cdc51*						
*Lcmt2*						
*Ccndbp1*						
*Thap1*						
*Gfra1*						
*Jak3*						

(b) Co-occurring gene alterations in *HBsAg^tg/0^and PML^−/−^HBsAg^tg/0^mice*				
Cell growth & signal	ROS pathway	Transcription & translation	Ubiquitination	Others
*Trib3*	*Duox2*	*Creb312*	*Fbxw9*	*Col6a3*
*Nanog*	*Gstcd*	*Nanog*	*Cyld*	*Ramp1*
*Cnksr1*	*Pon2*	*Pnrc1*	*Haus7*	*Sycp1*
*Larp7*		*Gtf3a*		*Ccdc112*
*Disc1*		*Dlx5*		*Slc5a6*
*Map3K9*		*Ubtf*		
		*Tceb2*		
		*Eif5*		

Loss of *PML* increased the mutation rates (> 50%) of the pathways, signaling molecules, or genes involved in DNA repair (*Fancc, Ercc1*, and *Pcna*), chromatin remodeling (*Arid5b*), cell growth (*Jak3, Cdk6*, and *Pdgfa*), lipid metabolism (*Glud1* and *Ugt1a family*), ubiquitination (*Ube4a, Usp21*, and *Siah1a*), membrane trafficking (*Pld5* and *Cpne1*), and ribosomal biogenesis (*Rrs1*). Based on our finding that *PML^−/−^* mice did not develop HCC, but did develop severe steatosis and proliferative disorders in multiple organs throughout their lifetime (supplementary Fig. S1), a predisposition for mutations in DNA repair genes did not appear to be a prerequisite for HCC initiation.

The knockdown of *PML* facilitated early mutations in *HBsAg*-transgenic liver cells. In the preneoplastic livers of *PML^−/−^HBsAg^tg/0^* mice by 3 months of age, we observed an exceptionally high prevalence (>50%) of mutations in the cancer drivers *Ctnnb1* (which encodes β-catenin of the Wnt signaling cascade), *Trp53* (which encodes p53 and maintains genomic integrity), *Cdk4* (which drives cell cycle progression) and *Mllt4* (which contributes to chromatin remodeling). These genes are commonly mutated in human HCCs and other solid tumors [28][29].

The mutation rate was lower in *PML^+/+^HBsAg^tg/0^* mice than in *PML^−/−^* and *PML^−/−^HBsAg^tg/0^* mice. However, in the presence of *PML*, albeit at a slow rate after two years, the *HBsAg* transgene still progressively induced frequent mutations (>50%) in genes of oxidative stress pathways (*Duox2, Gstcd*, and *Pon2*), cell growth signaling (*Nanog, Trib3, Larp7, Hck*, and *Disc1*), transcriptional factors (*Dlx5, Tceb2*, and *Ubtf*), metabolism (*Mdh1* and *Nudt9*), chromatin remodeling (*Smarca5*), and ubiquitination (*Haus7, Fbxw9*, and *Cyld*). The enrichment in oxidative stress-related gene mutations in *HBsAg*-transgenic mice corresponded to the long-term HBsAg-induced ER stress in the liver of human HBV carriers and subsequent HCC development very late in life [13][14].

The overall longitudinal mutational profiles of the life time in the livers of *PML^−/−^, PML^+/+^HBsAg^tg/0^* and *PML^−/−^HBsAg^tg/0^* mice revealed overlapping features, but also mutually exclusive portraits, which allowed us to correlate genotypes with phenotypic evolution. Many mutually exclusive mutations were previously shown to be involved in human cancer initiation and progression [28][29]. Here, *Ctnnb1, Cdk4, Trp53* and *Mllt4* were identified in early-onset HCCs of *PML^−/−^HBsAg^tg/0^* mice, and *Smarca5*, *Mdh1* and *Nudt9* were identified in late-onset HCCs of *PML^+/+^HBsAg^tg/0^* mice. Since the *PML* gene of *PML^+/+^HBsAg^tg/0^* mice was not mutated during the whole HBsAg-induced pathogenesis, spatiotemporal correlations of the restoration of PML with HCC progression and burnt-out steatosis after decline of HBsAg implicated that the late-onset, aggressive, angiogenic HCC in *PML^+/+^HBsAg^tg/0^* mice might be addicted to PML [30][31].

### Oncogenic addiction to *PML* in late-onset HBsAg-induced HCC progression in mice

**Figure 5:**
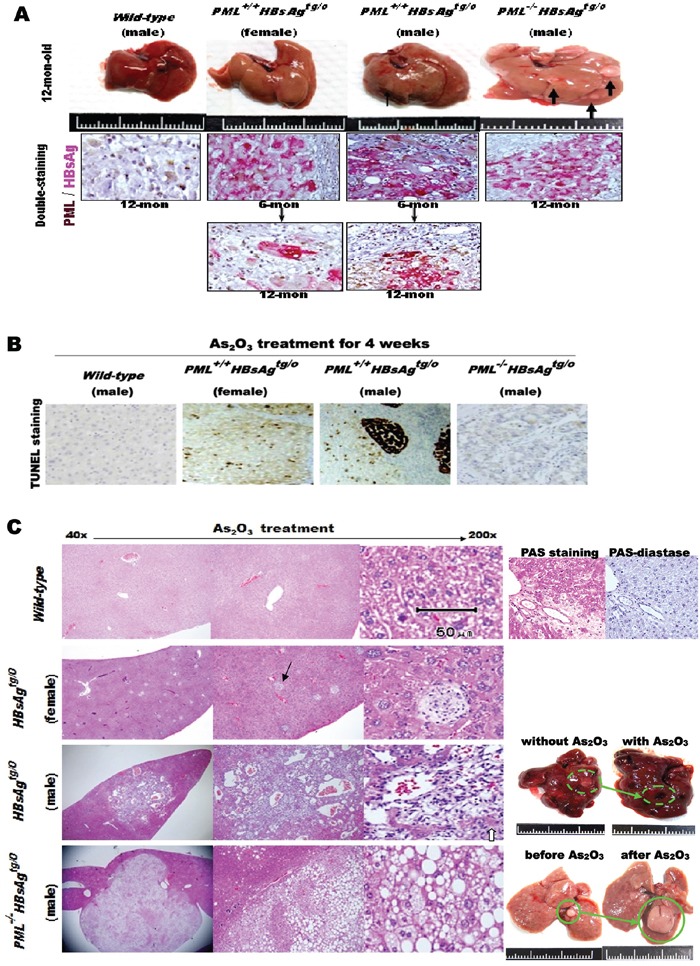
Effects of As_2_O_3_ on liver cell death in HBsAg-transgenic mice, with or without PML deletion **A.** Macroscopic analysis of representative livers from one-year-old *wild-type* (n=16), *PML^+/+^HBsAg^tg/0^* (n-36) and *PML^−/−^HBsAg^tg/0^* (n=24) mice before As_2_O_3_ treatment (*top row*), showing pale hepatomegaly and multiple small HCCs (thick black arrows) in the *PML^−/−^HBsAg^tg/0^* liver, and small adenoma (thin black arrow) in the *PML^+/+^HBsAg^tg/0^* male liver. Double-staining immunohistochemistry of PML and HBsAg in mouse liver sections (*lower panels*), showing that cytoplasmic HBsAg expression (pink) in *HBsAg*-transgenic mice is gradually lost over time. Concomitantly, nuclear PML (brown) is restored in the HBsAg-lost liver cells of *PML^+/+^HBsAg^tg/0^* mice. **B.** Representative TUNEL-stained sections from the livers of mice treated with As_2_O_3_ for 4 weeks. In *PML^+/+^HBsAg^tg/0^* mice, apoptosis (dark brown) occurred focally in females and confluently in males **C.** Representative histological images of H&E stained liver sections from mice one month after completion of As_2_O_3_ treatment showing glycogen accumulation (purple-magenta) (PAS-diastase (an enzyme that breaks down glycogen) as a control) in wild-type mice and multiple holes (black arrow) in *PML^+/+^HBsAg^tg/0^* mice, filled with inflammatory cell aggregates due to hepatocyte apoptosis in females, and large apoptotic areas with sinusoidal ectasia due to confluent hepatocyte death in males. Some residual hepatocytes (white arrow) remained within the sinusoidal ectasia. In addition to selective hepatocyte death in *PML^+/+^HBsAg^tg/0^* mice, there were also vast differences in liver fat accumulation and tumor-onset timing in the livers of *PML^+/+^HBsAg^tg/0^* and *PML^−/−^HBsAg^tg/0^* mice at one year of age.

As_2_O_3_ degrades PML *in vivo* and *in vitro*, mimicking *PML* loss in genetically engineered mice [32][33]. We thus treated one-year-old male and female *PML^+/+^*HBsAg**, *PML^−/−^HBsAg^tg/0^* and *wild-type* mice with intraperitoneal injections of As_2_O_3_ (3 mg/kg) or PBS (control) every two days for four weeks. Among the three genotypes of mice, only *PML^+/+^*HBsAg** mice showed increased levels of PML while HBsAg decayed (Figure 5A). Although slight body weight loss was noted in mice treated with As_2_O_3_, we observed no differences in physical activity, eating, or physical appearance between mice treated with this dose of As_2_O_3_ and mice treated with PBS (placebo control) in groups of the same genotype. After four weeks of As_2_O_3_ treatment, we performed *in-situ* TUNEL analysis on excised mouse livers. We detected an induction of focal and confluent apoptosis in the *PML^+/+^HBsAg^tg/0^* mice, but not *wild-type* or *PML^−/−^HBsAg^tg/0^* mice (Figure 5B). Liver pathology was also examined in the livers excised at one month after the completion of As_2_O_3_ treatment (Figure 5C). The livers of *wild-type* mice treated with As_2_O_3_ showed glycogen accumulation (predominantly clear cells positive to PAS-staining) with no hepatocyte degeneration or necrosis. The continuing growth of early-onset, fat-like HCC tumors was not inhibited in *PML^−/−^HBsAg^tg/0^* mice treated with As_2_O_3_, compared to those treated with PBS. In contrast, the severely dysplastic livers with microadenomas of *PML^+/+^HBsAg^tg/0^* male mice treated with As_2_O_3_ exhibited confluent necrotic areas with selective loss of hepatocytes and preservation of endothelial and mesenchymal cells, resulting in sinusoidal reticular ectacia development with inflammatory cell infiltration. However, the mildly dysplastic livers of *PML^+/+^HBsAg^tg/0^* female mice treated with As_2_O_3_ showed multifocal, small necrotic spots filled with inflammatory cell aggregates, similar to a clone-like clustering cell death. These differences were consistent with the finding that HBV-associated pathogenesis typically developed 3-5 months later in females than in males [23].

**Figure 6:**
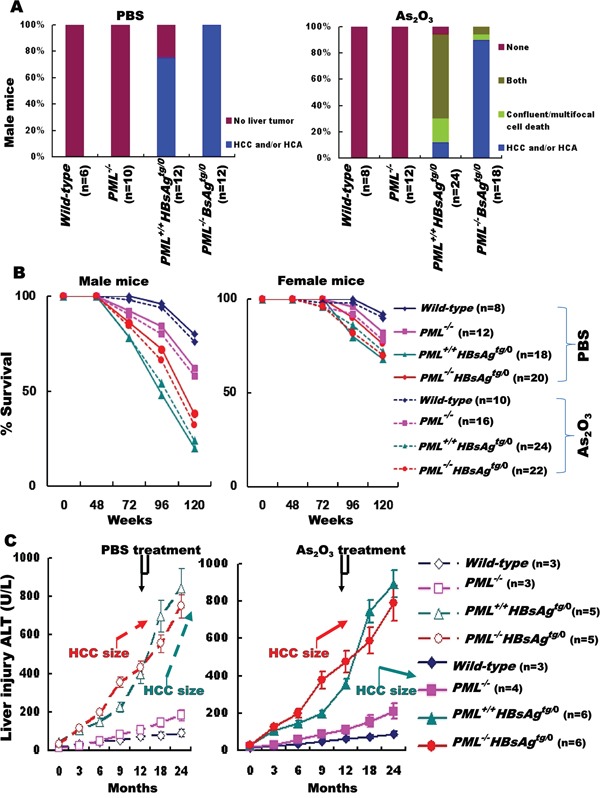
Outcomes of As_2_O_3_ treatment for HCC in HBsAg-transgenic mice with or without PML deletion **A.** Summary of histopathology performed on livers from one-year-old male mice with different genotypes (*wild-type*, *PML^−/−^*, *PML^+/+^HBsAg^tg/0^* and *PML^−/−^HBsAg^tg/0^*), one month after treatment with PBS (left) or As_2_O_3_ (right) **B.** Survival rates for male and female mice of various genotypes treated with (dashed lines) or without (solid lines) As_2_O_3_. **C.** Alanine aminotransferase (ALT) levels in surviving male mice (n=3-6) before, during and after PBS (left) or As_2_O_3_ (right) treatment (4-week window indicated at top).

The extent of As_2_O_3_-induced cell death in one-year-old *PML^+/+^HBsAg^tg/0^* mice reflected on decrease in the incidence of liver tumor development at 1.5 years of age (Figures 5C and 6A). The *HBsAg* transgene, but not the *PML* deletion, induced HCC and/or hepatocellular adenoma (HCA) and As_2_O_3_ treatment significantly increased hepatocyte death while decreasing HCC development only in *PML^+/+^HBsAg^tg/0^* mice. However, the tumor-inhibitory effect of As_2_O_3_ did not dramatically improve survival at two years of age (Figure 6B). Survival rates for *PML^+/+^HBsAg^tg/0^* mice with very late-onset angiogenic HCC responding to As_2_O_3_ were lower than for *PML^−/−^HBsAg^tg/0^* mice with very early-onset adipose-like HCC resistant to As_2_O_3_, suggesting that HCC without PML might have a relatively indolent, low-grade character. Indeed, the survival rate of *HBsAg*-transgenic mice with HCC was inversely correlated with the severity of long-term HBsAg-induced liver injury, but did not correspond to the HCC size (Figure 6C).

## DISCUSSION

Unlike 30% of the chronic HBV human carriers susceptible to infection-promoted immune-inflammatory processes of hepatitis, cirrhosis and/or carcinogenesis of the liver, the transgenic mice expressing the entire HBV genome, carrying the HBe or HBV-core protein, or producing infective HBV particles show no increased incidence of HCC and no pathological changes [34][35]. However, 80-100% of the *HBsAg*-transgenic and 40-80% of the *HBx*-transgenic mice under different promoter control developed oxidative stress and then a trabecular type of HCC in a male-predominant manner around 1.5-2 years of age, as observed in human HCCs [23][35]. Since HBsAg appears to be a dominant HBV onco-protein, the transgenic mice carrying *HBsAg* alone might provide insight into virus-host interactions and serve as animal models for evaluation of anti-HBV therapy [36]. In the present study, we found that the growth regulator PML, which can be upregulated by interferon or downregulated by As_2_O_3_ [33][37], acts as a physiological, reciprocal interaction partner of HBsAg. Also, PMLs spatiotemporally dose-dependent action affects the phenotypic evolution, tumorigenesis and lipid metabolism of *HBsAg*-transgenic mice.

PML was initially identified as a tumor suppressor that maintains genomic stability, activates the function of p53, inhibits the PI3K pathway [38] and is commonly degraded in virus infection and frequently lost in various types of cancer [15][16][39]. In contrast, PML has also been shown to behave as an oncogene or pro-survival factor that activates PPAR signaling and enhances fatty acid oxidation to maintain stem cell self-renewal and promote cancer cell survival [30][31][32][40][41][42][43]. In line with the two opposing activities of PML shown in chronic myelocytic leukemia and triple-negative breast carcinoma cells [15][26][40][41][42], we also found a dual role for PML in HBsAg-related pathogenesis. Loss of PML at the early phase predisposed to tumor development while presence of PML at the later phase enhanced metabolic reprogramming and tumor progression. Knockdown of *PML* increased genome instability and prompted *HBsAg*-transgenic mice to develop early-onset adipose-like HCCs. When HBsAg was lost and HBsAg-associated PML suppression was relieved, re-appearance of PML in later phase enhanced a metabolic shift from glycogen storage to lipolysis, which implicated increased energy availability for driving the angiogenic HCC progression. Thus, the reciprocal negative interaction observed with differential ratios between HBsAg and PML, which started with HBsAg^extensive^/PML_suppression_ and progressed to HBsAg^lost^/PML_re-appearance_, might represent a spectrum of liver diseases with different biological consequences in hepatocarcinogenesis, mutation landscapes and metabolic reprogramming.

Our findings also indicated that parts of the early HBsAg effects on genomic instability and steatosis might be related to degradation of PML, and the late manifestation of burnt-out steatosis during HBsAg-related HCC progression might reflect restoration of PML from the HBsAg-associated degradation due to decline of HBsAg. Previous *in vitro* studies demonstrated that proteasome-mediated degradation of PML could be triggered by ectopically overexpressed SIAH-1/2 [20]. However, we found that strong expression of SIAH-1/2 in human HBV-related HCC specimens did not correlate with downregulation of PML (Figure 1), suggesting that PML is not a physiological substrate of SIAH-1/2 and the supposed negative interaction between PML and SIAH-1/2 may not account for the tumorigenic effect of HBsAg. Since the *PML* gene is rarely mutated in solid tumors, and it is not epigenetically silenced, whether the mechanism involved in HBsAg-induced PML degradation is associated with hepatocarcinogenesis remains to be determined [15][16]. Nevertheless, given that PML deficiency resulted in altered metabolism from a glycogenotic state to enhanced adipogenesis and even obesity in *HBsAg*-transgenic mice (Figure 2 and 5), PML could be the long-sought link between chronic HBV-infection and metabolic reprogramming toward HCC development [43][44][45]. However, it remains unclear how to reconcile PML's tumor suppressive effects on cancer growth versus its oncogenic activities in enhancing lipid metabolism in different cellular and pathological contexts [13][40][41][42]. Interferon-mediated upregulation of *PML* transcription and As_2_O_3_-mediated PML protein degradation have both been shown to suppress growth and induce apoptosis in human HCC cell lines and animal HCC models [33][37]. However, treatment with either interferon or As_2_O_3_ was not effective in patients with advanced HCC in clinical trials [46][47]. This raises concern about the timing of PML-targeting treatments for HBV-related HCC. We showed that pharmacological inhibition of PML by treating mice with As_2_O_3_ only selectively killed the long-term HBsAg-transformed liver cells that had low HBsAg levels and rising PML levels. Our findings suggested that it might be counterproductive to use As_2_O_3_ prematurely for preventing early-onset HBV-related hepatocarcinogenesis, because in that state (HBsAg^high^/PML_low_), PML functions as a tumor suppressor. However, As_2_O_3_ is effective when given in the late phase of HBV-related HCC progression (HBsAg^low^/PML_high_), because it inhibits PML oncogenic pro-survival effects. On the contrary, interferon should be given early in the PML suppression phase.

Our observations might explain why both interferon and As_2_O_3_ treatment failed to show therapeutic benefits on unselected patients with HCC in clinical trials. Thus, our study emphasizes the need to profile HBsAg and PML expression in liver cells prior to embarking on therapy. Since the interaction of PML and HBsAg could be a predictive factor, reanalysis of the previous interferon and As_2_O_3_ trials is warranted in HBV-related HCC patients that are stratified by the HBsAg/PML expression ratio. Nevertheless, the corresponding survival gain might be lower than expected. Our study showed that As_2_O_3_ decreased the size and incidence of HBsAg^low^/PML_high_ HCCs but it did not stop the progression of HBsAg-induced liver injury that is also a determining survival factor. On the other hand, based on its effect on promoting tumor progression by enhancing fatty acid oxidation, PML could also be a prognostic factor of cancers that undergo metabolic reprogramming. Consistent with the clinical findings that loss or low levels of PML reflected less aggressive characters in cancer metabolism and better survival [40][41], *PML^−/−^HBsAg^tg/0^* mice that developed very early-onset fat-accumulating HCC by one year of age lived as long as *PML^+/+^HBsAg^tg/0^* mice that developed late-onset fat-burning HCC after 1.5 years (Figure 6C).

Resulting from the reciprocally dynamic interactions between HBsAg and PML, the dual activities of PML, the subsequent divergent mutations in growth signaling pathways, and the different phenotypic and metabolic evolutions driven by high and low HBsAg/PML ratios, inter-tumor and intra-tumor heterogeneity should occur with time. Based on this heterogeneity, single-target therapy for HBV-related HCC would be expected, at most, to produce modest response rates with short durations. These insights into the diverse, pleiotropic roles of PML at different stages of HBsAg-induced HCC development and progression could lead to novel therapeutic interventions. For example, combinatorial therapies could be developed to hit multiple targets, including HBsAg (with anti-sense approaches), PML-nuclear bodies (with As_2_O_3_ and retinoic acid applications), HBsAg-induced ER stress (with phenylbutyrate), PML-deficient pathways involved in DNA repair (with olaparib), and alterations in lipid metabolism (with bezafibrate). This strategy may lead to therapies with synthetic lethality in both early-onset and late-onset HBV-related HCC and therapies that prevent HBV-associated pathogenesis from liver injury and metabolic reprogramming.

## MATERIALS AND METHODS

### Ethics statement

This study strictly adhered to the recommendations in the Guide for the Care and Use of Laboratory Animals of the National Institutes of Health. The animals were handled according to approved guidelines by the Institutional Animal Care and Use Committee. The animal protocols were approved by the Institutional Review Board of the Koo Foundation Sun Yat-Sen Cancer Center, Taipei, Taiwan. The mice were housed and maintained at the National Laboratory Animal Center in Taiwan, under institutionally approved conditions. The collection of tissue samples followed the recommendations given in the Declaration of Helsinki and its amendments.

### Mice

*PML*^−/−^ mice (129/SV-*Pml^tm/Ppp^* from the National Cancer Institute, USA) [26] were crossed with liver-specific *HBsAg*-transgenic mice (C57BL/6J-Tg(Alb1HBV)44Bri/J from Jackson Lab, USA) [23]. The resulting offspring were then crossed to produce different mouse genotypes, including *wild-type, PML^+/−^, PML^−/−^, PML^+/+^HBsAg^tg/0^, PML^+/−^HBsAg^tg/0^*, and *PML^−/−^HBsAg^tg/0^*, after several generations. These strains, with similar genetic backgrounds (129/SV x C57BL/6J), were confirmed by whole exome sequencing, PCR genotyping and immunohistochemistry. About 20-50 mice of each genotype were sacrificed at 1-24 months of age for macroscopic and microscopic analyses of the liver, pancreas, spleen, kidneys, brain, intestines, heart and lungs.

Previously, we found that there were no differences in the pathogenesis, incidence, timing, sex disparity and survival of HCC development between the parental*PML^+/+^HBsAg*-transgenic C57BL/6J strain and the *PML^+/+^HBsAg*-transgenic mice derived from crossing C57BL/6J with 129/SV background [21]. Similar to the parental *PML*-knockout 129/SV strain, our genetically mixed *PML^−/−^* mice (129/SV x C57BL/6J) did not develop malignancy spontaneously but were prone to proliferative disorders.

### Histopathology, *in-situ* TUNEL staining, and immunohistochemistry

Tissues were fixed in formalin, dehydrated, and embedded in paraffin. Deparaffinized sections (4-5 μm) were treated with 3% H_2_O_2_ to block endogenous peroxidase activity, then immersed in boiling 0.01% citric acid (pH 6.0) in a microwave oven for 15 min to enhance antigen retrieval. After cooling, some sections were rehydrated with PBS and stained with hematoxylin and eosin (H&E), the terminal deoxynucleotidyl transferase dUTP nick end labeling (TUNEL) reagent (R&D systems), or periodic acid-schiff (PAS) or PAS-diastase (Sigma-Aldrich) for liver glycogen according to the manufacturer's instructions. Other sections were treated with the following antibodies: anti-PML (Santa Cruz, 1:25 dilution), anti-HBsAg (Dako, 1;100 dilution), or antibodies raised against the E3 ligases, seven in absentia homolog-1 and -2 (Cell Signalling, 1:50 dilution). Immunohistochemistry was performed with the ABC Kit (Dako). An isotype antibody was also used as a control. Immunohistochemical analyses of archival paraffin blocks derived from patients was approved by the Institutional Review Board of the Koo Foundation Sun Yat-Sen Cancer Center, Taipei, Taiwan.

### Drug treatment

As_2_O_3_ (arsenic trioxide) directly binds PML, which promotes covalent modification of PML by the small ubiquitin-like modifier (SUMO). PML SUMOlyation is followed by ubiquitylation and proteasome-mediated degradation [33]. To test the effect of pharmacological PML inhibition, As_2_O_3_ or an equal volume of PBS was injected intraperitoneally into treatment or control mice, respectively. Symptoms of toxicity were evaluated once each week, including changes in skin pigmentation, hair loss, physical activity, food consumption, lethargy and body weight. One month after completing the drug treatment, mouse livers were grossly examined and sectioned for pathology. In addition, approximately 60-100 μl of blood from surviving male mice (n=3-6) were collected from the tail vein. Alanine aminotransferase (ALT), an indicator of the severity of liver injury, was measured in the Clinical Laboratory Medicine at the Koo Foundation Sun Yat-Sen Cancer Center using standard procedures.

### Whole exome library preparation and sequencing

Flash-frozen livers were digested overnight with proteinase K (Bioline) and genomic DNA was extracted with a DNeasy Tissue kit (Qiagen). Genomic DNA integrity was assessed by electrophoresis on 1% agarose gels. DNA quantity and quality were examined by analyzing samples on a High Sensitivity DNA Bioanalyzer chip (Agilent Technologies, Santa Clara, CA). Double-stranded DNA concentrations were determined with a Qubit Hi-sensitivity DNA assay kit (Life Technologies). Exome enrichment was performed with the Agilent Sure Select XT Mouse All Exon kit. According to the manufacturer's recommended protocol, exomes were quantitated and qualified with Qubit and Bioanalyzer chip assays. The enriched libraries were then subjected to sequencing with the Illumina GAIIx or the Illumina HiSeq2500 (Illumina), according to the manufacturer's protocols. To check the read quality after sequencing, nucleotides with low quality scores (<13) were first trimmed from the sequence reads. Overall, DNA sequencing generated a mean target coverage of 164×, and a mean of 95.5% of the target sequence was covered to a depth of at least 10×. Both coverage and depth were similar between cohorts. The analysis followed the *best practices* recommended by the Broad Institute. Read sequences were aligned to a mouse reference genome. The exome single nucleotide polymorphism (SNP) calls were produced with the GATK SNP calling pipeline [48]. Variant annotation was conducted with snpEff to add information, such as the name of the gene that harbored the variant, the effect of the mutation (missense variant, synonymous variant, etc.), and information from the SNP database, dbSNP [49][50].

## SUPPLEMENTARY FIGURES AND TABLES





## Abbreviations

HBV: hepatitis B virus
HBsAg: HBV surface antigen
HCC: hepatocellular carcinoma
PML: Promyelocytic Leukemia
ER: endoplasmic reticulum
*HBsAg^tg/o^*: *^HBsAg^*-transgenic mouse
ALT: alanine aminotransferase
