# Supplementary material for: Dual oncogenic and tumor suppressor roles of the promyelocytic leukemia gene in hepatocarcinogenesis associated with hepatitis B virus surface antigen
Source: Oncotarget. 2016 Apr 6;7(19):28393–407. doi: 10.18632/oncotarget.8613 (PMC5053734; doi:10.18632/oncotarget.8613)
Supplement: Supplementary file 1 [file oncotarget-07-28393-s001.pdf]

Dual oncogenic and tumor suppressor roles of the promyelocytic leukemia gene in hepatocarcinogenesis associated with hepatitis B virus surface antigen

SUPPLEMENTARY FIGURE AND TABLE

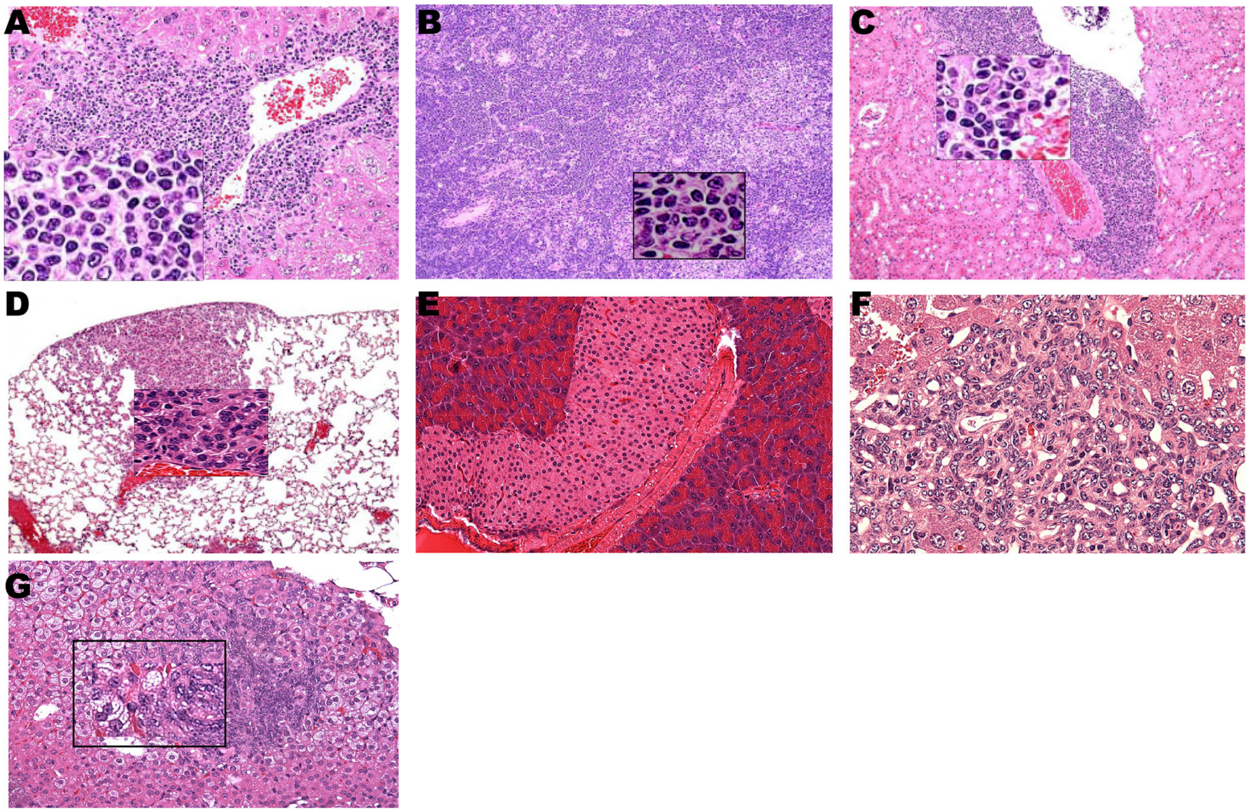

Supplementary Figure S1: *PML* knock-out mice are prone to developing proliferative disorders. A small proportion of *PML*$^{-/-}$ mice (about 12%) and *PML*$^{-/-}$*HBsAg*$^{tg/0}$ mice (about 18%), but no *HBsAg*$^{tg/0}$ mice, were found to have **A.** pleiomorphic neoplastic lymphoid cells with prominent nucleoli in the liver, **B.** predominantly neoplastic lymphoid cells that formed sheets in the mesenteric lymph nodes, **C.** neoplastic lymphoid cells in the kidney, **D.** focal bonchio-alveolar adenoma in the lung, **E.** pancreatic islet hyperplasia, **F.** bile duct proliferation, and/or **G.** spindle cell tumors in the adrenal gland.

Supplementary File S1:

See Supplementary File 1
